# Factors Affecting the Quality of Histopathologic Specimens Obtained via Small Intestinal Endoscopic Biopsy in Dogs and Cats

**DOI:** 10.1111/jvim.70059

**Published:** 2025-03-08

**Authors:** Ko Nakashima, Kazuhiro Kojima, Yoshinori Takeuchi, Manami Ito, Isao Matsumoto, Takahiro Ushigusa, Hiroshi Ohta, Kazuyuki Uchida

**Affiliations:** ^1^ Japan Small Animal Medical Center Saitama Japan; ^2^ Department of Veterinary Pathology, Graduate School of Agricultural and Life Sciences The University of Tokyo Tokyo Japan; ^3^ Department of Data Science, School of Data Science Yokohama City University Yokohama Kanagawa Japan; ^4^ Yokohama Animal Medical Center Kannai Animal Clinic Kanagawa Japan; ^5^ Companion Animal Internal Medicine, Department of Companion Animal Clinical Sciences, School of Veterinary Medicine Rakuno Gakuen University Ebetsu Hokkaido Japan

**Keywords:** duodenum, filter paper‐fixation, generalized estimating equations, histopathologic adequacy, ileum

## Abstract

**Background:**

The factors affecting the quality of histopathologic specimens obtained via small intestinal endoscopic biopsy (SIEB) remain unclear.

**Hypothesis/Objectives:**

To identify factors related to the quality of histopathologic specimens obtained via SIEB.

**Animals:**

Histopathologic duodenal and ileal specimens were obtained from 116 dogs and 38 cats that underwent SIEB for diagnostic purposes.

**Methods:**

This retrospective study analyzed 3354 individual histopathologic specimens scored using the grading system of histopathologic adequacy (GSHA). A lower GSHA score indicates lower quality specimens. Univariate and multivariate ordinal logistic models were used to assess the relationship between the GSHA score of the specimens and various explanatory factors, including fixation method, biopsy forceps size, biopsy site, and histopathologic diagnosis. The generalized estimating equation method was used to account for the clustering of specimens among animals.

**Results:**

Multivariate models using the specimens showed that filter paper fixation of endoscopic samples resulted in a higher GSHA score than floating fixation in both dogs (ordinal odds ratio [OR]: 0.19; 95% confidence interval [CI]: 0.15–0.25) and cats (ordinal OR: 0.19; 95% CI: 0.13–0.29). In dogs, the scores were lower for duodenal specimens than for ileal specimens and for specimens obtained using smaller forceps. In cats, the scores were lower for ileal specimens than for duodenal specimens and for older animals.

**Conclusion and Clinical Importance:**

The quality of histopathologic specimens obtained via SIEB is influenced by the fixation method. Additionally, other factors differ between dogs and cats. These results contribute to improved SIEB practices in veterinary medicine.

**Abbreviations**
CI: confidence interval
GEE: generalized estimating equations
GSHA: grading system of histopathologic adequacy
OR: odds ratio
SIEB: small intestinal endoscopic biopsy

## 1 Introduction

Gastrointestinal endoscopic biopsies play pivotal roles in the diagnosis of gastrointestinal mucosal disease in dogs and cats. However, the maximum information cannot be obtained if diagnostic samples are not obtained [1]. It has been suggested that multiple endoscopic biopsy samples of good quality are necessary for pathologists to make an accurate diagnosis, and conversely, that it may not be possible to make an accurate diagnosis based on poor quality samples [2]. Although the American College of Veterinary Internal Medicine Consensus Statement and review article provide detailed information about endoscopic biopsy and specimen handling procedures [1, 3], the actual histopathologic specimens obtained via endoscopic biopsies of dogs and cats exhibit considerable variability, ranging from low‐quality specimens revealing only villi to high‐quality specimens affording a comprehensive view of the entire mucosa. This substantial variability in specimen quality poses a significant challenge in histopathologic diagnosis. Several factors associated with high‐quality histopathologic specimens have been reported, such as tissue fixation using nonabsorbent sponge or cucumber slices [4], using 2.8‐mm channel biopsy forceps with larger cups rather than smaller cups [5], and opting for a teaching hospital rather than private practice [6]. Furthermore, it is more difficult to obtain an adequate sample from the small intestine than the stomach [4, 5, 6]. However, information regarding factors influencing the quality of histopathologic specimens is limited to the stomach and duodenum, while the quality of ileal specimens has not been investigated. In addition, no study has performed a comprehensive multivariate analysis of the factors related to the quality of histopathologic specimens obtained via small intestinal endoscopic biopsy.

We hypothesized that the quality of histopathologic specimens obtained via small intestinal endoscopic biopsy is affected by multiple factors, such as fixation method, biopsy forceps size, biopsy site, and histopathologic diagnosis. The aim of the present study was to report the percentage of scores based on the grading system of histopathologic adequacy (GSHA) [5] and identify key factors related to the quality of histopathologic specimens obtained via small intestinal endoscopic biopsy in dogs and cats.

## 2 Materials and Methods

### 2.1 Collection of Histopathologic Slides

We retrospectively reviewed histopathologic slides of dogs and cats that underwent endoscopic small intestinal biopsy. Medical records and histopathologic slides were collected from the database of the Japan Small Animal Medical Center, a small animal referral hospital, from January 2017 to April 2018. The inclusion criteria were: (1) small intestinal biopsies obtained endoscopically from dogs and cats with a history of gastrointestinal signs; (2) availability of medical records and histopathologic slides. Dogs and cats with focal small intestinal lesions such as adenocarcinoma or eosinophilic sclerosing fibroplasia were excluded. Written informed consent for data collection and usage in this study was obtained from the owners of each dog and cat.

### 2.2 Endoscopic Biopsy Sample Collection and Tissue Handling

**FIGURE 1:**
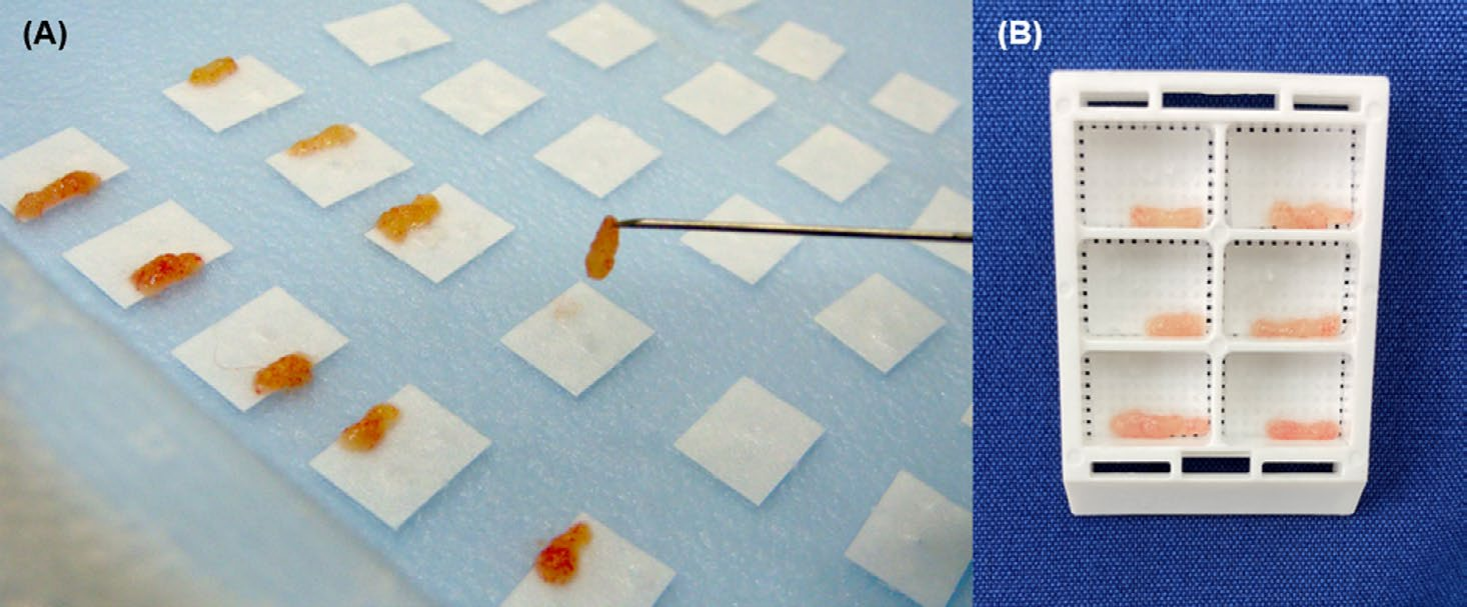
(A) Endoscopic biopsy tissues were gently mounted and oriented with the mucosal side up and submucosal side down on a saline‐moisturized filter paper using a 25G needle. (B) Tissue specimens mounted on filter papers encased in plastic cassettes.

A single well‐trained endoscopist with more than 16 years of experience in endoscopy (Ko Nakashima) performed all endoscopic examinations and biopsies on patients under general anesthesia. The endoscopes used to obtain the specimens were the Olympus VQ‐8143B, Olympus GIF‐XP290N, or Olympus SIF‐H290N; the appropriate endoscope was chosen based on the patient's body weight and small intestinal diameter. The biopsy forceps were either the Olympus FB‐54Q‐1 (alligator cup biopsy forceps for a 2.8‐mm channel) or the Olympus VH‐142‐B54 (alligator cup biopsy forceps for a 2.0‐mm channel). All endoscopic biopsies were performed under direct visualization of the mucosa. The tissue specimens obtained via endoscopic biopsy measured 4 to 8 mm in length and 1 to 2 mm in depth and were thus deemed sufficient for pathological examination. Each tissue specimen was gently detached from the forceps using a 25G needle and processed using one of two different fixation methods. Some tissue specimens were gently mounted and oriented with the mucosal side up and the submucosal side down on a saline‐moisturized filter paper, encased in plastic cassettes, and immersed in 10% formalin (Figure 1A,B). The other tissue specimens were directly immersed in 10% formalin while floating free and were then fixed. The specimens were submitted to the histopathologic laboratory by the next day after collection, processed routinely, embedded in paraffin wax, cut into 4‐μm‐thick paraffin sections, and stained with hematoxylin and eosin. All sampling methods were performed with signed written consent from all owners.

### 2.3 Histopathologic Adequacy and Diagnosis

The GSHA score of each specimen was evaluated by the same pathologist (IM), as previously described [5]. The GSHA comprised a 4‐point scale in which a score of 1 indicated an inadequate specimen (tissue that had only villi or subvillus lamina propria, but not both), a score of 2 indicated a marginal specimen (tissue that had at least one villus plus subvillus lamina propria, but did not clearly have the full thickness of the subvillus lamina propria extending to the muscularis mucosa), a score of 3 indicated an adequate specimen (tissue that had at least three villi and subvillus lamina propria that extended to the mucosa‐muscularis mucosa border, whether or not it included the muscularis mucosa), and a score of 4 indicated a superior specimen (tissue that had at least seven villi and had subvillus lamina propria that extended to the mucosa‐muscularis mucosa border, whether or not it included the muscularis mucosa) [5].

The histopathologic diagnosis was evaluated by two pathologists (Isao Matsumoto and Kazuyuki Uchida). The diagnosis of chronic enteritis and severe lymphangiectasia was based on the histopathologic standards for endoscopic biopsy samples developed by the World Small Animal Veterinary Association Gastrointestinal Standardization Group [7]. Only dogs were assessed for lymphangiectasia, as this condition is rare in cats. Small‐cell and large‐cell lymphoma were diagnosed histopathologically, as previously described [8, 9].

### 2.4 Statistical Analysis

We compared the GSHA score in accordance with several categorical factors. Ordinal logistic regression analysis was used to investigate the key factors influencing the GSHA score of the histopathologic specimens.

The potential association between the GSHA score and the following explanatory variables was explored: fixation method (floating‐fixation or filter paper‐fixation), species (dog or cat), sex (male or female), weight (< 5.0 kg, 5.0–10.0 kg, or > 10.0 kg), age (< 6 years old, 6–12 years old, or > 12 years old), biopsy site (duodenum or ileum), biopsy forceps size (forceps for the 2.0‐ or 2.8‐mm channel), and histopathologic diagnosis (chronic enteritis, small‐cell lymphoma, or large‐cell lymphoma; presence or absence of severe lymphangiectasia). The GSHA score was defined as an ordinal variable. Univariate and multivariate ordinal logistic models were used to assess the relationship between the GSHA score of histopathologic specimens and each explanatory factor. Ordinal odds ratio (OR) estimates were interpreted as the OR for changing the GSHA score by one unit; that is, an ordinal OR estimate < 1 meant that the factor was associated with high‐quality histopathologic specimens, whereas an ordinal OR estimate > 1 indicated a factor that adversely affected the quality of histopathologic specimens. The lower estimates of ordinal OR indicated the higher quality of the histopathologic specimens. The generalized estimating equation method was used to account for the clustering of specimens among animals [10]. Statistical analyses were performed using the multgee package (version 1.9.0) in R statistical software (version 4.2.3; R Foundation for Statistical Computing, Vienna, Austria).

## 3 Results

A total of 3354 individual pieces of histopathologic specimens were analyzed, comprising 2521 specimens from 116 dogs and 833 from 38 cats. The median ages of the dogs and cats were 9.4 years (range, 1.0–14.6 years) and 11.4 years (range, 2.0–15.3 years), respectively. The median body weights of the dogs and cats were 5.6 kg (range, 1.5–30.8 kg) and 4.3 kg (range, 2.9–7.4 kg), respectively. Table 1 summarizes the number of histopathologic specimens categorized by species, biopsy site, fixation method, forceps size, and histopathologic diagnosis.

**TABLE 1:** Number of histopathologic specimens categorized by species, biopsy site, fixation method, forceps size, and histopathologic diagnosis.

Variables	Number of histopathologic specimens (number of animals)	
	Dog	Cat
Biopsy sites		
Duodenum	1638 (116)	552 (38)
Ileum	883 (112)	281 (36)
Fixation methods		
Filter paper	2138 (116)	694 (38)
Floating	383 (64)	139 (24)
Forceps		
For 2.8 mm channel	1807 (94)	37 (4)
For 2.2 mm channel	714 (48)	796 (38)
Histopathologic diagnosis		
Severe intestinal lympangiectasia	510 (24)	NA
Chronic enteritis	1832 (83)	452 (21)
Small‐cell lymphoma	346 (16)	311 (14)
Large‐cell lymphoma	343 (17)	70 (3)
Total	2521 (116)	833 (38)

Abbreviation: NA, not available.

**FIGURE 2:**
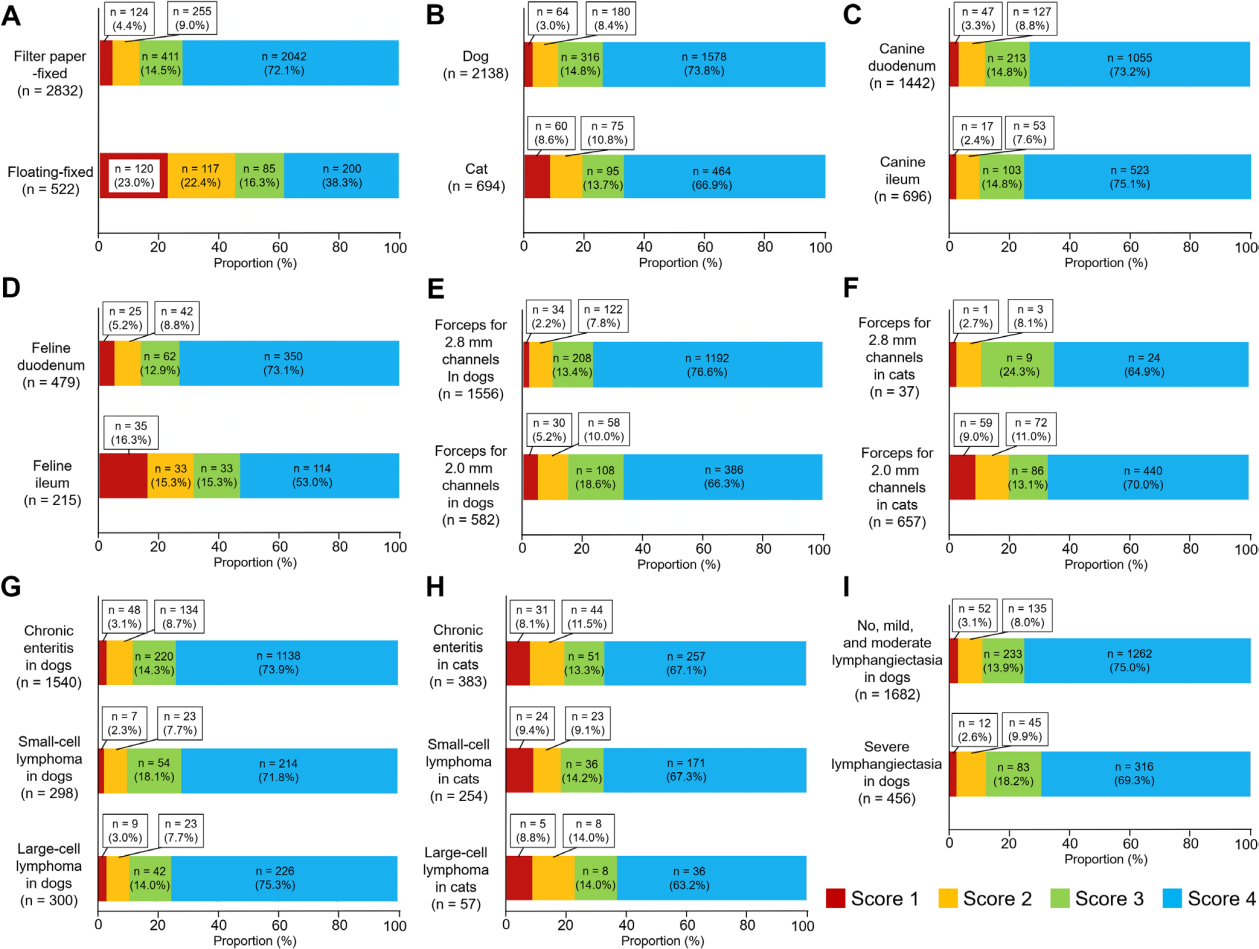
The proportions of the GSHA score of (A) filter paper‐fixed or floating‐fixed specimens in dogs and cats, (B) filter paper‐fixed specimens in dogs and in cats, filter paper‐fixed duodenal or ileal specimens in (C) dogs and (D) cats, filter paper‐fixed specimens obtained using forceps for the 2.8‐mm or 2.0‐mm channels in (E) dogs and (F) cats, filter paper‐fixed specimens with a histopathological diagnosis of chronic enteropathy, small‐cell lymphoma, or large‐cell lymphoma in (G) dogs and (H) cats, and (I) filter paper‐fixed specimens in dogs with and without severe lymphangiectasia. GSHA, grading system of histopathologic adequacy.

Figure 2 showed the proportions of GSHA scores of each explanatory variable, and the results of univariate ordinal logistic models for each explanatory variable are summarized in Table 2. Filter paper‐fixed specimens exhibited a higher GSHA score than floating‐fixed specimens (Figure 2A). A univariate ordinal logistic model revealed that filter paper fixation was associated with higher GSHA scores than floating fixation (ordinal OR: 0.2; 95% confidence interval [CI]: 0.16–0.25). Among the filter paper‐fixed specimens, the biopsy sites were associated with the GSHA scores of feline specimens, with the GSHA score being lower for specimens from the ileum compared with the duodenum (Figure 2D; ordinal OR: 2.67; 95% CI: 1.89–3.77). Similarly, the forceps size was associated with the GSHA score of canine specimens, as specimens obtained using forceps for 2.8‐mm channels tended to have a higher GSHA score than those obtained using forceps for 2.0‐mm channels (Figure 2E; ordinal OR: 0.64; 95% CI: 0.47–0.87). There were no clear associations between the GSHA score and other variables in univariate models. In the multivariate ordinal logistic model adjusted for sex, age, and body weight, in addition to the fixation method and forceps size, the biopsy site was an independent factor associated with the GSHA score of canine specimens (Table 3). Unlike feline specimens, the canine duodenal specimens showed a reduced GSHA score compared with the canine ileal specimens. In the feline specimens, in addition to the fixation method and biopsy site, older age was an independent factor that decreased the GSHA score (Table 4).

**TABLE 2:** Univariate ordinal logistic models for each explanatory variable.

Variables	Category	Number of histopathologic specimens	Ordinal OR	95% CI
Fixation methods	Floating	522	Reference	
	Filter paper	2832	0.20	0.16–0.25
Among filter paper‐fixation samples				
Species	Cat	694	Reference	
	Dog	2138	0.84	0.58–1.24
Biopsy sites in dogs	Duodenum	1442	Reference	
	Ileum	696	0.89	0.71–1.12
Biopsy sites in cats	Duodenum	479	Reference	
	Ileum	215	2.67	1.89–3.77
Forceps in dogs	For 2.0‐mm channel	582	Reference	
	For 2.8‐mm channel	1556	0.63	0.42–0.95
Forceps in cats	For 2.0‐mm channel	657	Reference	
	For 2.8‐mm channel	37	0.85	0.50–1.44
Histopathologic diagnosis in dogs	Chronic enteritis	1540	Reference	
	Small‐cell lymphoma	298	1.17	0.77–1.78
	Large‐cell lymphoma	300	0.92	0.53–1.60
Histopathologic diagnosis in cats	Chronic enteritis	383	Reference	
	Small‐cell lymphoma	254	0.98	0.45–2.12
	Large‐cell lymphoma	57	1.23	0.64–2.36
Severe lymphangiectasia in dogs	Absent	1682	Reference	
	Present	456	1.38	0.87–2.18

*Note:* An ordinal OR < 1 meant that the factor is associated with high‐quality histopathologic specimens, while an ordinal OR > 1 indicates a factor that adversely affects the quality of histopathologic specimens. The lower estimates of ordinal OR indicated the higher quality of the histopathologic specimens.

Abbreviations: CI, confidence interval; OR, odds ratio.

**TABLE 3:** Multivariate ordinal logistic model for canine specimens.

Variable	Category	Number of histopathologic specimens	Ordinal OR	95% CI
Sex	Female	1266	Reference	
	Male	1255	1.02	0.77–1.35
Age	< 6 years old	477	Reference	
	6–12 years old	1605	1.37	0.97–1.94
	> 12 years old	439	1.04	0.63–1.70
Weight	< 5.0 kg	1099	Reference	
	5.0–10.0 kg	1006	1.19	0.80–1.77
	> 10.0 kg	416	1.17	0.74–1.87
Fixation methods	Floating	383	Reference	
	Filter paper	2138	0.19	0.15–0.25
Biopsy sites	Duodenum	1638	Reference	
	Ileum	883	0.73	0.58–0.90
Forceps	For 2.0‐mm channel	714	Reference	
	For 2.8‐mm channel	1807	0.52	0.37–0.74
Histopathologic diagnosis	Chronic enteritis	1832	Reference	
	Small‐cell lymphoma	346	1.13	0.72–1.76
	Large‐cell lymphoma	343	0.87	0.54–1.41
Severe lymphangiectasia	Absent	2011	Reference	
	Present	510	1.31	0.87–1.97

*Note:* An ordinal OR < 1 meant that the factor is associated with high‐quality histopathologic specimens, while an ordinal OR > 1 indicates a factor that adversely affects the quality of histopathologic specimens. The lower estimates of ordinal OR indicated the higher quality of the histopathologic specimens.

Abbreviations: CI, confidence interval; OR, odds ratio.

**TABLE 4:** Multivariate ordinal logistic model for feline specimens.

Variable	Category	Number of histopathologic specimens	Ordinal OR	95% CI
Sex	Female	353	Reference	
	Male	480	1.10	0.57–2.15
Age	< 6 years old	89	Reference	
	6–12 years old	415	3.39	1.76–6.53
	> 12 years old	329	5.13	2.65–9.93
Weight	< 5.0 kg	652	Reference	
	5.0–10.0 kg	181	0.97	0.55–1.74
Fixation methods	Floating	139	Reference	
	Filter paper	694	0.19	0.13–0.29
Biopsy sites	Duodenum	552	Reference	
	Ileum	281	2.46	1.69–3.59
Forceps	For 2.0‐mm channel	796	Reference	
	For 2.8‐mm channel	37	1.47	0.75–2.90
Histopathologic diagnosis	Chronic enteritis	452	Reference	
	Small‐cell lymphoma	311	0.90	0.44–1.87
	Large‐cell lymphoma	70	1.13	0.50–2.56

*Note:* An ordinal OR < 1 meant that the factor is associated with high‐quality histopathologic specimens, while an ordinal OR > 1 indicates a factor that adversely affects the quality of histopathologic specimens. The lower estimates of ordinal OR indicated the higher quality of the histopathologic specimens.

Abbreviations: CI, confidence interval; OR, odds ratio.

## 4 Discussion

The current study identified several factors that may influence the quality of histopathologic specimens obtained from small intestinal endoscopic biopsies in dogs and cats through multivariate analysis. The analysis of specimens revealed that the fixation method was a key factor that affected the quality of histopathologic specimens. In addition, the independent factors that decreased the quality of histopathologic specimens were the duodenal biopsy site and smaller forceps size in dogs, and the ileal biopsy site and older age in cats.

In the present study, the filter paper‐fixation method was superior to the floating‐fixation method in obtaining high‐quality histopathologic specimens. To the best of the authors' knowledge, no research to date has clarified the effect of the filter paper‐fixation method on the quality of endoscopic biopsy specimens in dogs and cats. With the floating‐fixation method, it may be difficult for laboratory staff to determine the mucosal surface of small samples, and fixation in a bent state may make it difficult to prepare a histopathologic specimen with a cross‐section perpendicular to the mucosa. In contrast, in the filter paper‐fixation method, the mucosal samples were affixed to the filter paper with the villi facing up, making it easy to determine the orientation of the samples. Although a previous study reported better results with the fixation method using cucumber slices or sponges than with the floating‐fixation method [4], filter paper has advantages over these fixation surfaces in that it takes less time for preparation, costs less, and is a uniform material, making it a practical choice for routine use.

Interestingly, the biopsy site affected the quality of biopsy specimens in the present study; that is, ileal specimens were better quality than duodenal specimens obtained from dogs, while the reverse was true for specimens obtained from cats. The cause of this difference in quality based on the biopsy site is not clear but may be influenced by the thickness of the mucosa or the handleability of the endoscope. As the duodenal mucosa is thicker than the ileal mucosa in dogs [11, 12], it may not be biopsied completely or may not stand vertically on the filter paper. In the attainment of cat ileal specimens in the present study, the forceps often pinched not only the mucosa but also the submucosa, and only fragmented mucosa was extracted when the forceps were withdrawn. Thus, the collected ileal samples were fragile and the mucosal surface was difficult to see. This may be because the ileal mucosa of cats is too thin [13]. Furthermore, in the present study, the cat ileum often contracted due to peristalsis during endoscopy and did not expand properly when air was pumped in, which may have made this region difficult to biopsy. As ileal biopsies are becoming increasingly common because of discrepancies between duodenal and ileal histopathologic results [14, 15, 16], further investigations using improved techniques are warranted to improve the quality of histopathologic specimens of the ileum in cats.

The quality of the histopathologic specimens from dogs in the present study was better when collected using forceps for 2.8‐mm channels with a larger cup size than with forceps for 2.0‐mm channels, which is in line with the findings of a previous study [5]. Therefore, forceps for a 2.8‐mm channel are recommended if an endoscope with a 2.8‐mm channel can be inserted into the small intestine in dogs. In the present study, an endoscope with a 2.8‐mm channel was infrequently used in cats due to the narrow diameter of the small intestine. Consequently, it is believed that the results of this study cannot conclusively determine the effect of forceps size in cats.

In the present study, older age was associated with a decrease in the quality of histopathologic specimens, especially in feline specimens. While the exact cause remains unclear, it is possible that age‐related changes in the mucosal architecture might contribute to this observation. Further studies are needed to elucidate the impact of age on small intestinal biopsy specimen quality.

Previous review articles have recommended the attainment of 10–15 good quality endoscopic biopsy samples for the canine duodenum and six good quality endoscopic biopsy samples for the feline duodenum [1]. Furthermore, the same review articles recommended the attainment of 3–5 good quality endoscopic biopsy samples for the canine and feline ileum, although the exact number remains uncertain [1]. As fewer good quality histopathologic specimens are obtained when using the floating‐fixation method, obtaining samples from the canine duodenum, obtaining samples from the feline ileum, and using forceps for 2.0‐mm channels, it may be acceptable to collect more than the abovementioned recommended number of samples in these instances. However, differences in histopathologic diagnosis did not affect the quality of histopathologic specimens in the present study. Therefore, the number of biopsy samples may not need to be changed according to the suspected diseases or severity of enteritis.

Our study had some limitations. First, all biopsy forceps used in this study were reusable instruments. Further research is needed to determine whether the number of times a biopsy forceps is used affects the quality of the specimens. Second, as several staff members were involved in placing collected tissues on the filter paper and paraffin embedding, staff differences may have affected the test results. Third, data on the size, weight, or artifacts of endoscopic biopsy samples were not collected. Fourth, as 97 of the 116 (83.6%) dogs weighed less than 10 kg, further studies are needed to determine the factors affecting the quality of small intestinal endoscopic biopsy samples in larger dogs.

In conclusion, the filter paper –fixation method is more effective in creating good‐quality specimens than the floating –fixation method. As different factors may affect the quality of histopathologic specimens, it may be necessary to consider increasing the number of biopsy samples on a case‐by‐case basis.

## Disclosure

Authors declare no off‐label use of antimicrobials.

## Ethics Statement

Authors declare no Institutional Animal Care and Use Committee or other approval was needed. Authors declare human ethics approval was not needed.

## Conflicts of Interest

The authors declare no conflicts of interest.
